# Regucalcin in the Tumor Microenvironment: From Intracellular Tumor Suppression to Putative Extracellular Signaling

**DOI:** 10.3390/cancers18162655

**Published:** 2026-08-17

**Authors:** Naoomi Tominaga

**Affiliations:** Graduate School of Medicine, Yamaguchi University, 1-1-1 Minami-Kogushi, Ube 755-8505, Yamaguchi, Japan; ntominag@yamaguchi-u.ac.jp

**Keywords:** regucalcin (RGN), tumor suppressor, tumor microenvironment (TME), signaling molecule, cancer therapy

## Abstract

Regucalcin (RGN), also known as senescence marker protein-30, is a calcium-binding protein without an EF-hand motif that regulates intracellular calcium homeostasis and Ca^2+^-dependent enzyme activity. Its expression is reduced in tumor tissue relative to matched normal tissue across several cancer types, and restoring RGN in cancer cell lines suppresses proliferation, migration, and invasion through cell cycle arrest, reduced expression of matrix metalloproteinases and epithelial–mesenchymal transition regulators, and transcriptional reprogramming involving p53, p21, Rb, c-myc, and β-catenin. Attention has more recently turned to RGN outside the cell. RGN is detectable in serum and interstitial fluid, and recombinant RGN applied to cancer cells reproduces much of the suppression seen upon intracellular overexpression, which has prompted the proposal that extracellular RGN acts within the tumor microenvironment (TME). That proposal, however, rests on evidence that remains incomplete. RGN carries no classical signal peptide and its route of release is undefined; it has been reported in extracellular vesicle preparations but has not been shown to be a bona fide vesicular cargo; no receptor or proximal binding partner has been identified; and its effects on the non-malignant compartment of the TME—fibroblasts, immune cells, and endothelium—have not been tested directly. Here, we review the tumor-suppressive activity of RGN, distinguish demonstrated findings from inferred ones, and outline the experiments required to determine whether extracellular RGN constitutes a signaling axis in the TME or a pharmacological property of a recombinant protein.

## Simple Summary

Regucalcin (RGN) is a calcium-binding protein that helps cells keep their internal calcium balance. Its levels are lower in many cancer tissues than in the corresponding normal tissues, and raising RGN levels in cancer cells slows their growth, movement, and invasion. RGN is also found outside cells, in blood, and in the fluid around tissues, and RGN added to the outside of cancer cells suppresses their growth. This raises a question that has not been answered: how does RGN get out of the cell, and what does it act on once it is there? RGN has been reported in preparations of extracellular vesicles, the small membrane packets that cells use to exchange material, but it has not been shown that RGN is truly carried inside them. In this review, we summarize what is known about RGN in cancer, separate the findings that are established from those that are assumed, and set out the experiments needed to decide whether extracellular RGN is a genuine signal within the tumor microenvironment.

## 1. Introduction

The tumor microenvironment (TME) is now understood not as a passive scaffold but as a compartment in which cancer cells, stromal cells, immune cells, and the extracellular matrix exchange signals that shape the course of disease [1,2]. Much of the work on this exchange has concentrated on secreted factors with defined receptors and on extracellular vesicles (EVs) as carriers of protein and RNA cargo [3,4,5]. Proteins that were long regarded as strictly intracellular and that are found outside the cell without an obvious means of getting there occupy a more difficult position in this framework. Regucalcin (RGN) is one such protein.

RGN was identified in 1978 as a calcium-binding protein that, unlike calmodulin, lacks the EF-hand motif [6,7] and was independently characterized as senescence marker protein-30 (SMP30) [8,9]. Its established functions are intracellular: it regulates Ca^2+^-dependent enzyme activity, participates in the oxidative stress response, and modulates transcription relevant to cell proliferation [10,11]. Over the past decade, a consistent body of work has shown that RGN expression is reduced in tumor tissue relative to normal tissue in several cancer types, and that restoring RGN in cancer cell lines suppresses proliferation, migration, and invasion [6,12,13]. These findings have been reviewed comprehensively elsewhere [6,13] and support the designation of RGN as a tumor suppressor.

A second and more recent set of observations is less easily accommodated. RGN is detectable in serum and in interstitial fluid, and recombinant RGN applied to the outside of cancer cells reproduces much of the suppression seen when RGN is overexpressed within them [13,14,15,16]. This has led to the proposal that RGN acts extracellularly within the TME [13]. The proposal is reasonable, but the intervening steps are not established. RGN carries no classical signal peptide, and no export pathway has been assigned to it; it has been reported in EV preparations [17,18], though without the orthogonal evidence that current standards require before a protein is called a vesicular cargo [19]; no receptor or cell-surface binding partner has been identified; and the effect of extracellular RGN on the non-malignant cells that constitute the TME has not been tested. Whether extracellular RGN is a physiological signal within the TME or an experimentally useful property of a recombinant protein, therefore, remains open.

This review has two aims. The first is to summarize the intracellular tumor-suppressive activity of RGN and the evidence for its extracellular activity, drawing on the primary literature. The second, which distinguishes it from earlier reviews, is to separate what has been demonstrated from what has been inferred. We give particular attention to the route by which RGN reaches the extracellular space, to the question of whether it is genuinely associated with EVs, and to the experiments that would settle these points. Our purpose is not to diminish the case for RGN as a therapeutic target, which is supported by consistent preclinical data, but to identify the gaps whose resolution determines whether that case rests on a signaling axis within the TME or on a pharmacological effect.

## 2. Regucalcin: Structure, Regulation, and Cellular Functions

Regucalcin (RGN) was initially identified in 1978 as a novel calcium-binding protein. In contrast to calmodulin and other calcium-binding proteins, RGN possesses a unique structure that lacks the EF-hand motif [11,20,21]. Its nomenclature is derived from its function as a “regulator of calcium,” modulating various Ca^2+^-dependent enzyme activities in hepatocytes [22,23]. RGN is also known as senescence marker protein-30 (SMP30) [8,9]. The RGN gene (rgn) is located on the X chromosome and is conserved in more than 15 vertebrate species, including humans, and in some invertebrates [10,24]. The expression of the RGN gene is modulated by the transcription factor RGPR-p117 [25,26]. This factor translocates to the nucleus in a protein kinase C-dependent manner and directly binds to the nuclear factor I consensus sequence on the RGN promoter, thereby enhancing its transcription [25]. The high level of conservation indicates that RGN has crucial physiological functions that have been maintained throughout evolution [11,24]. Through this mechanism, RGN regulates numerous Ca^2+^-dependent enzymes and signaling pathways, thereby maintaining balanced intracellular signaling [27].

Moreover, RGN functions as a defense against oxidative stress [11,28]. Hepatic RGN declines with age, and its identification as senescence marker protein-30 derives from this observation [8,29]. It is associated with a wide array of pathological conditions, including aging [28], chronic inflammatory states linked to NF-κB activation [30], and lifestyle-related illnesses [13]. Through its antioxidant properties, RGN contributes to the protection of cells and tissues and has been implicated in energy metabolism, protein synthesis, and intercellular signaling [11]. In rodents, RGN is expressed predominantly in the liver, with markedly lower levels in most other tissues [21]. In human tissues, expression is high in both the liver and the kidney [20]. The tissue distribution of RGN bears on the interpretation of circulating RGN and is returned to in Section 7.4.

## 3. Intracellular Regucalcin Suppresses Tumor Cell Behavior

Recent studies have demonstrated that RGN exhibits diverse molecular regulatory mechanisms that inhibit cancer cell proliferation, metastasis, and invasion [6]. Specifically, experiments involving the forced expression of the RGN gene in various human cancer cell lines, such as HepG2 liver cancer cells [31], MDA-MB-231 breast cancer cells [32], pancreatic cancer cells [6], and prostate cancer cells [33], consistently showed a significant reduction in cell proliferation. For instance, in 3-(4,5-dimethylthiazol-2-yl)-2,5-diphenyltetrazolium bromide (MTT) assays and 5-bromo-2’-deoxyuridine (BrdU) incorporation analyses, cell proliferation rates 48 h after treatment were reduced by 34–67% compared to controls [6], with this inhibitory effect likely involving cell cycle arrest. Flow cytometry analysis revealed that RGN-overexpressing cells underwent cell cycle arrest at either the G1 or G2/M phase, indicating the regulation of cell division and proliferation [34].

Moreover, RGN diminishes the metastatic and invasive potential of malignant tumor cells. It specifically suppresses the expression of extracellular matrix-degrading enzymes (including MMP-3, MMP-7, and MMP-9) and modulates EMT-related molecules such as E-cadherin and N-cadherin, in association with Wnt/β-catenin signaling [34]. Transwell and wound healing assays showed a decrease of approximately 50% in migratory and invasive activities. This suggests a mechanism by which RGN effectively reduces the risk of local invasion and distant metastasis by cancer cells. In a bone metastasis model of prostate cancer, the introduction of RGN resulted in a decreased capacity for bone metastasis and even induced dormancy of tumor cells, highlighting a novel molecular basis for its metastasis-suppressive effects [33,35].

At the molecular level, RGN facilitates the induction of tumor-suppressive transcription factors, such as p53, Rb, p21, and p27, while controlling cancer-related signaling networks through the transcriptional suppression of cancer-related genes (such as ras, c-myc, Stat3, β-catenin, and NF-κB) [6,36]. In non-small cell lung cancer cells, RGN has been reported to suppress Cyclin D1 expression through regulation of histone deacetylase 4 [37]. RGN also inhibits the Wnt/β-catenin pathway by reducing the nuclear accumulation of β-catenin [6]; since Wnt/β-catenin signaling drives the transcription of matrix metalloproteinases [38], this provides a plausible route by which RGN restrains extracellular matrix remodeling. Chromatin Immunoprecipitation (ChIP) assays and immunofluorescence analyses demonstrated that RGN translocates into the nucleus and associates with promoter regions of cancer-related genes, participating in chromatin modification [39].

In summary, RGN exhibits complex antitumor effects by regulating the cell cycle, inhibiting extracellular matrix degradation and EMT, and reprogramming transcriptional networks. Through these mechanisms, RGN significantly impacts cancer cell proliferation and metastasis. These findings provide insights into the molecular pathology of cancer and the development of new cancer therapies.

Recent studies have demonstrated that RGN expression is reduced in cancer cells, leading to enhanced tumor formation, to metastasis, and to increased cell proliferation, underscoring its potential as a tumor suppressor gene (Table 1) [6,12,40].

## 4. Regucalcin Expression in Human Tumors and Clinical Correlates

RGN expression differs markedly between normal and tumor tissue in humans. Reduced RGN expression in tumor relative to normal tissue, and its association with prognosis, has been reported across major cancer types, including hepatocellular carcinoma and lung, breast, colorectal, prostate, and renal cancers.

At the protein level, immunohistochemical staining and western blotting have similarly demonstrated that RGN protein expression is diminished by approximately 20–70% in tumor tissues compared to normal tissues across various cancers, including liver (HCC), colon, pancreatic, and breast cancers [6]. A consistent trend of expression loss in advanced cancers and cases with distant metastasis has been reported [32,49].

The mechanisms responsible for the loss of RGN expression in tumors have received less attention than its consequences. Several transcription factors have been reported to bind the regucalcin promoter and enhance its transcription, including AP-1, NF1-A1, and RGPR-p117 in the rat gene [25,50], and hypoxia-inducible factor-1α, which occupies a hypoxia-response element in the promoter [26]. Additional factors, including β-catenin, NF-κB, and STAT3, have been listed as regulators of the regucalcin gene in review articles [6,50], although the primary evidence for direct promoter binding is less complete. Beyond transcription, two further layers have been described in human tumors. In non-small cell lung cancer, methylome and transcriptome analysis of tumor and matched tumor-free tissue from the same donors showed hypermethylation of CpG loci at the regucalcin transcription start site, inversely correlated with transcript level [51]. In lung squamous cell carcinoma, RGN was placed within a computationally predicted competing endogenous RNA network involving miR-203a-3p and two long non-coding RNAs, which suggests a post-transcriptional layer of regulation that has not yet been tested experimentally [52].

Two considerations limit what can be concluded. Several of the transcription factors reported to act on the regucalcin gene—β-catenin, NF-κB, STAT3, and HIF-1α—are among the pathways that RGN is itself reported to suppress (Section 3), so an association between low RGN and activation of these pathways does not establish which is upstream. Nor has any study tested whether reversing a specific upstream lesion, such as promoter methylation, restores RGN expression and alters the malignant phenotype. That RGN is lost in tumors is well documented; whether that loss drives transformation or follows from it remains undetermined.

From a functional standpoint, tumor-suppressive effects have been demonstrated using in vivo models in which RGN expression was restored or introduced. For example, the introduction of RGN in a mouse model of prostate cancer bone metastasis suppressed the progression of bone metastatic lesions [33]. Additionally, it has been reported to reduce the risk of recurrence by inducing dormancy in bone marrow tumor cells [35].

Moreover, it has been suggested that RGN expression is relatively maintained in the early stages of malignant tumors, which has been proposed as a potential auxiliary biomarker for tumor staging and early detection [36].

## 5. Extracellular Regucalcin

RGN has been detected outside the cell, and exogenously applied RGN is active on cancer cells. This section summarizes those observations; their interpretation is examined in Section 7. Exogenous RGN has been applied in vitro at concentrations that are consistent across studies [15,16,40,41,48,53]. The concentration of RGN in the tumor interstitium has not been measured, however, so it is not known how the experimental conditions relate to those encountered by cells in tissue. In vitro studies have demonstrated that the administration of exogenous RGN, such as recombinant RGN or synthetic RGN peptides, results in a range of antitumor activities [15]. These include the inhibition of proliferation and migration, the reduction of bone metastasis activity, and the suppression of invasive potential in human cancer cell lines derived from the liver, breast, pancreas, prostate, osteosarcoma, ovary, and glioblastoma [13]. These effects are accompanied by mechanisms such as induction of cell cycle arrest at the G1 and G2/M phases and by reduced activity of major signal transduction pathways, including PI3K/Akt, MAPK, and Wnt/β-catenin. Exogenous RGN has not been reported to induce apoptosis; the studies that examined cell death state explicitly that it was unaffected [15,16,41,50]. The suppression of growth by extracellular RGN is therefore cytostatic rather than cytotoxic, so far as has been tested. The antitumor effect of extracellular RGN is comparable to that observed when RGN is overexpressed intracellularly. In every such experiment, however, the recombinant protein was applied to cells that retained their endogenous RGN, so it has not been established that the effect originates outside the cell (Section 7.3). Extracellular RGN exposure suppresses EGFR-driven signaling, induces p53, Rb, and p21, and reduces c-myc and NF-κB activity. These are downstream readouts. Whether they follow from engagement of a cell-surface receptor, as is generally assumed, has not been determined (Section 7.4). Whether extracellular RGN also acts on the non-malignant cells of the TME—fibroblasts, immune cells, and endothelium—has been proposed but not tested; the available data derive almost entirely from cancer cell lines in monoculture (Section 7.6). The presence of RGN in the extracellular space has prompted two lines of translational interest. The concentration of RGN in serum is being investigated as a potential biomarker for diagnosis, staging, and recurrence monitoring in cancer patients. Furthermore, the use of exogenous RGN as a novel antitumor therapeutic strategy, such as adjunct therapy or local administration, is under exploration. Extracellular RGN therefore presents a coherent set of observations—detection outside the cell, reproducible suppression of cancer cell behavior on exogenous application—without a mechanism connecting them. Whether these observations describe a signaling axis operating within the TME or the pharmacological behavior of a recombinant protein in culture is the question addressed in Section 7.

## 6. Extracellular Vesicles as a Candidate Route for Regucalcin Release

RGN carries no classical signal peptide, and no export pathway has been assigned to it. Its presence in the extracellular space therefore requires an explanation. EVs are one candidate, and RGN has been reported in EV preparations. This section outlines what EVs are, in the limited respects relevant to that possibility, and reviews the reports in question.

Extracellular vesicles (EVs) are lipid bilayer-enclosed vesicular structures secreted by nearly all eukaryotic cells under physiological and pathological conditions [54]. EVs encapsulate a diverse array of biomolecules, including proteins, lipids, DNA, messenger RNA (mRNA), and microRNA (miRNA) [3,5]. EVs are small lipid-bilayer-bound vesicles secreted by cells and are primarily classified into three subtypes: exosomes, microvesicles, and apoptotic bodies [19]. However, in biological samples and clinical specimens, overlaps between subtypes and challenges in identification frequently arise due to the limitations of isolation technologies and analytical accuracy. Consequently, in instances where precise specification is challenging, it is advisable to use the overarching term “EVs”.

EVs are found in high abundance in circulating body fluids, including plasma [55], urine [56], saliva [57], and cerebrospinal fluid [58]. In contrast to normal cells, cancer cells markedly enhance EV secretion. For example, a study demonstrated that human cancer cells, such as medulloblastoma, secrete between 13,400 and 25,300 EVs per cell over a 48-h period, whereas normal fibroblasts secrete only 3800 to 6200 EVs. If RGN is released within EVs, tumor cells would be the principal source in the TME.

There is limited literature concerning regucalcin and EVs [17,18]. Recent studies have suggested the potential presence of a small amount of RGN in EVs. In research conducted by Arakawa et al., it was observed that while RGN protein levels in the liver decreased in an ODS rat model deficient in ascorbic acid, there was an increase in RGN levels within EVs [17]. Additionally, the study reported an elevation in acute-phase proteins, such as haptoglobin and ASGPR1, within EVs and activation of the Stat3 pathway, indicating a possible association with inflammatory responses and liver injury markers [17]. However, these findings are primarily derived from animal models, necessitating further investigation to elucidate the secretion of RGN into EVs and its functional significance in humans. It is crucial to exercise caution regarding EV purification methodologies, as polymer precipitation techniques applied to serum often result in the co-precipitation of abundant serum proteins, leading to notably low-purity EVs [59]. Consequently, it remains uncertain whether regucalcin is an intrinsic component of EVs or merely a co-precipitated contaminant.

## 7. Unresolved Questions in Extracellular Regucalcin Biology

**Figure 1:**
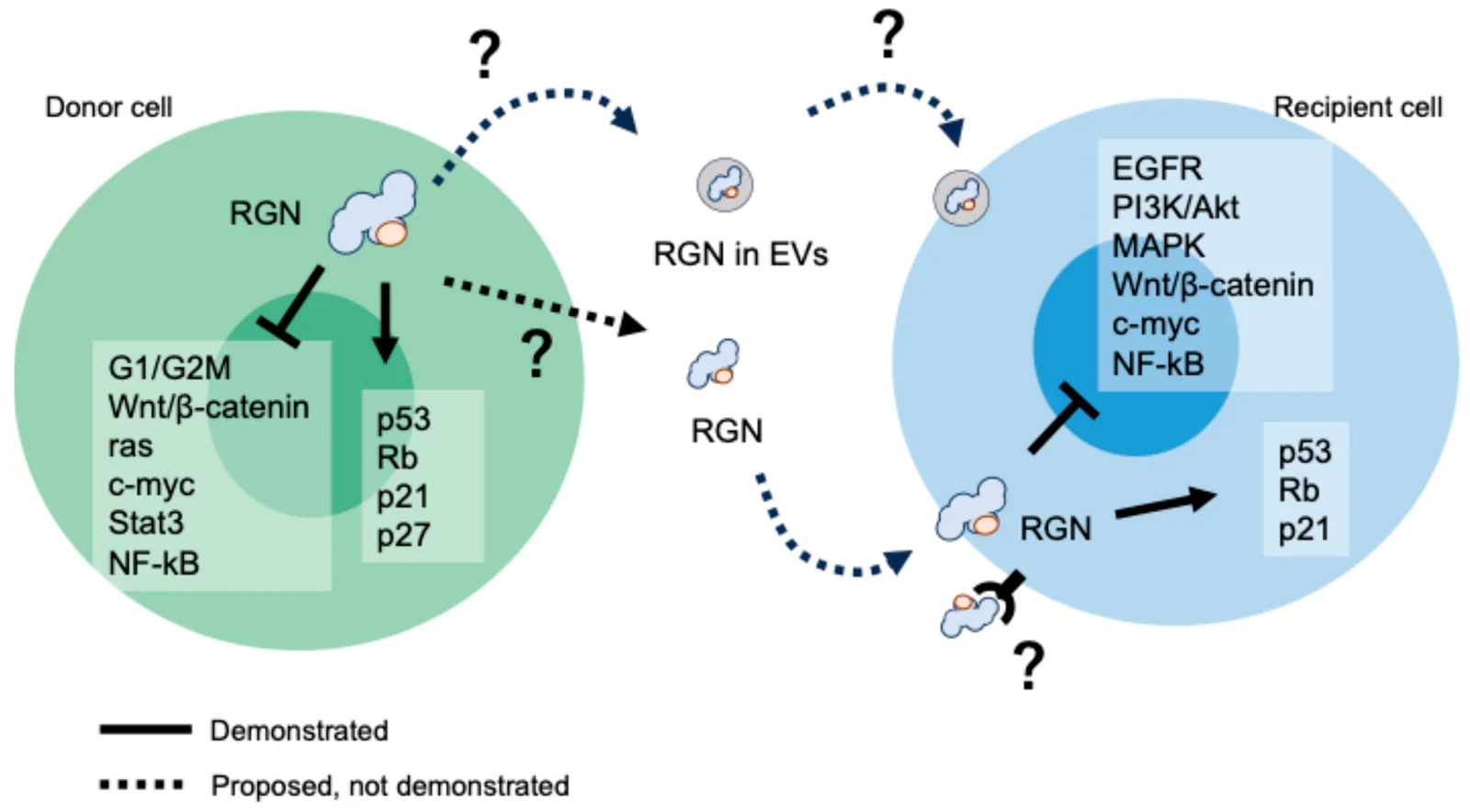
**Intracellular and extracellular regucalcin: demonstrated and proposed steps. Donor cell (left):** intracellular regucalcin suppresses cell cycle progression at G1 and G2/M and the Wnt/β-catenin pathway; represses transcription of ras, c-myc, Stat3, β-catenin, and NF-κB; and induces p53, Rb, p21, and p27. **Extracellular compartment (center):** Regucalcin is detected in serum, interstitial fluid, and extracellular vesicle preparations. The route by which it reaches this compartment is undetermined: regucalcin carries no classical signal peptide, and unconventional secretion, packaging into extracellular vesicles, and passive release from damaged cells have not been tested. Detection in a vesicle preparation is equally consistent with vesicular cargo and with co-isolation of the soluble protein. **Recipient cell (right):** Extracellular regucalcin suppresses EGF-driven signaling, reduces PI3K/Akt, MAPK, and Wnt/β-catenin activity, lowers c-myc and NF-κB, and induces p53, Rb, and p21. These are downstream readouts. The proximal event is undetermined: regucalcin has been proposed to engage binding sites on the plasma membrane and, alternatively or in addition, to be internalized, but neither has been demonstrated. **Line types:** Solid, supported by direct experimental observation; dashed with a question mark, proposed but not demonstrated. Blunt-ended connectors denote suppression; arrowheads denote induction.

The evidence summarized above supports a tumor-suppressive activity for RGN applied to cancer cells from the outside. It does not yet establish how RGN reaches that compartment, what it engages once there, or whether the concentrations achieved experimentally are attained in tissue. These gaps are not peripheral; they determine whether extracellular RGN is a signaling axis within the TME or a pharmacological effect of a recombinant protein. We set them out explicitly, since their resolution defines the near-term research agenda. Figure 1 summarizes the intracellular activity of regucalcin, the routes by which the protein might reach the extracellular space, and the routes by which it might act on a recipient cell, distinguishing steps that have been demonstrated from those that have been proposed. Table 2 summarizes the current evidentiary status of the principal claims concerning RGN in cancer, distinguishing what has been demonstrated from what remains inferred.

### 7.1. The Secreted Form of RGN Has Not Been Identified

RGN is described as a cytosolic protein that also localizes to the nucleus, mitochondria, and plasma membrane [11]. It is not, on current annotation, equipped with a classical N-terminal signal peptide, and no dedicated export machinery has been assigned to it. Yet RGN protein is detectable in serum and interstitial fluid, and exogenously applied RGN is biologically active on cancer cells [13,16,60]. These observations are difficult to reconcile without invoking one of three possibilities: unconventional (signal peptide-independent) secretion; packaging into EVs; or passive release from damaged or dying cells. The three are not mutually exclusive, and they carry different implications. Active secretion would place RGN within a regulated intercellular signaling circuit. Passive release would make extracellular RGN a consequence of tissue injury rather than a mechanism of tumor suppression, while leaving the therapeutic observations intact but reframing their biological meaning. To our knowledge, no study has directly compared these routes for RGN.

### 7.2. EV Association: Cargo or Co-Isolate?

The reports described in Section 6 [17,18], together with the cataloguing of RGN in Vesiclepedia, where human RGN is recorded in extracellular vesicle preparations from thirteen sample types, nine of them cancer cell lines of brain, breast, colorectal, kidney, hematological, lung, melanoma, ovarian, and pancreatic origin [61,62], share a single limitation: none was designed to test whether RGN is a genuine EV cargo. In each, RGN appears as a protein identified within an EV-enriched fraction, rather than as a protein demonstrated to be vesicle-associated.

The distinction is not pedantic. Polymer-based precipitation and single-step ultracentrifugation of biofluids co-enrich abundant soluble proteins and lipoprotein particles alongside vesicles [59], and MISEV2023 accordingly requires orthogonal evidence before a protein is assigned to EVs [19]. Vesiclepedia, for its part, catalogues proteins reported in EV studies without adjudicating whether each is a bona fide cargo. On the available data, therefore, the presence of RGN in an EV preparation remains equally consistent with co-isolation of the soluble protein. RGN occupies a position shared by many proteins in the EV literature: repeatedly detected, but never specifically tested.

Whether this is resolved matters mechanistically. A soluble ligand and an EV-borne cargo reach the recipient cell by different routes—receptor engagement at the plasma membrane versus vesicle uptake and intracellular delivery—and would be addressed by different therapeutic strategies. The experiments required to discriminate between them are established and are not technically demanding: density-gradient separation with parallel quantification of RGN in vesicular and non-vesicular fractions; protease protection to distinguish luminal from surface localization; and single-vesicle analysis. None has been reported for RGN.

The gap is therefore not one of conflicting data but of absent experiments. RGN has been identified in EV preparations from multiple sources, including tumor cells, and it is biologically active on cancer cells when applied extracellularly. What has not been determined is whether these two observations are connected—that is, whether the RGN that acts on cancer cells is delivered by vesicles, is present as a soluble protein, or both. Until this is established, the participation of RGN in EV-mediated crosstalk within the TME cannot be asserted.

### 7.3. Whether the Extracellular Effect Requires the Intracellular Pool Has Not Been Tested

In the published experiments, recombinant regucalcin has been applied to cancer cell lines that retain their endogenous RGN [15,16,40,41,48,53]. No study has combined exogenous application with deletion or knockdown of the endogenous gene. The suppression observed therefore cannot be assigned unambiguously to an event at the cell surface. Two accounts remain open. In the first, extracellular RGN engages the plasma membrane and initiates signaling from outside the cell. In the second, it is taken up and adds to the intracellular pool, in which case the exogenous experiments reproduce overexpression by a different route.

The second account has received less consideration than it warrants. The phenotype produced by exogenous RGN closely resembles that produced by intracellular overexpression (Section 3). Convergence of this degree is what would be expected if both manipulations act on the same intracellular pool. It does not exclude a surface receptor, but neither does it require one.

The available pharmacological data do not resolve the question. In several of these studies, the suppression produced by exogenous RGN was not increased by co-treatment with inhibitors of MEK, protein kinase C, phosphatidylinositol 3-kinase, calcium influx, or transcription [15,41,53]; in others it was not reversed by them [16,40]. The absence of additivity has been interpreted as evidence that extracellular RGN acts on the same intracellular pathways as those inhibitors. That interpretation is reasonable, but it is compatible with either account: a protein that is internalized would engage those pathways directly, and a protein acting at a receptor would engage them at one remove. The experiments were not designed to distinguish the two.

Discriminating between the accounts is not technically demanding. Applying recombinant RGN to cells in which RGN has been deleted or depleted would establish whether an endogenous pool is required for the response; retention of suppression in such cells would identify the effect as genuinely extracellular. Tracking labelled recombinant RGN, with surface-bound and internalized protein distinguished experimentally, would show whether uptake occurs at all. Restricting the protein to the cell surface, by immobilization or by conjugation to a carrier that cannot be internalized, would test whether engagement at the membrane is sufficient. None of these experiments has been reported.

### 7.4. The Proximal Target of Extracellular RGN Has Not Been Identified

Extracellular RGN has been reported to suppress EGF-driven signaling in glioblastoma cells [6,40] and to inhibit growth, migration, and adhesion in metastatic prostate cancer cells [16]. Downstream consequences—reduced PI3K/Akt, MAPK, and Wnt/β-catenin activity, induction of p53, Rb, and p21—are described. What is missing is the proximal event. A working model has nevertheless been advanced. Extracellular regucalcin has been proposed to bind putative regucalcin-binding sites on the plasma membrane and to transmit signals from there, and, alternatively or in addition, to be internalized following that binding [6,13,41]. The proposal has an empirical antecedent: ^125^I-labelled regucalcin binds isolated rat liver plasma membranes, in the presence or absence of Ca^2+^, and raises (Ca^2+^-Mg^2+^)-ATPase activity in that preparation, an effect that was not modified by GTPγS, glucagon, or norepinephrine and was concluded not to operate through a G protein-coupled receptor [63]. What this establishes is that regucalcin can associate with a plasma membrane and alter the activity of a membrane enzyme in a cell-free system from normal liver. The molecular identity of the site, its presence on cancer cells, and its relation to the growth suppression described above remain undetermined, and the internalization step has been introduced as a hypothesis rather than demonstrated experimentally. No receptor has been identified, no binding assay has been reported in the cancer cells on which extracellular RGN is active, and it has not been excluded that RGN acts by sequestering ligands in the medium rather than by engaging the cell. Cell-surface binding assays, chemical crosslinking with proteomic identification, and loss-of-function screening in responsive lines are the obvious approaches. Without such data, the term “extracellular RGN signaling” remains a description of outcome rather than of mechanism.

### 7.5. The Origin of Circulating RGN Is Confounded by Hepatic Expression

Proposals to use serum RGN as a diagnostic or prognostic biomarker must contend with a confounder that has received little attention. RGN is expressed at high levels in the liver, and hepatic RGN declines with age and under oxidative stress [28,29]. Circulating RGN may therefore reflect hepatocyte mass, hepatocyte differentiation state, or hepatocellular injury, independently of any tumor-derived contribution. In patients with hepatocellular carcinoma—the setting in which RGN has been studied most extensively—these variables are not separable without deliberate control. Interpreting a change in serum RGN as a tumor signal requires, at minimum, adjustment for liver function and comparison against non-malignant liver disease. We are not aware of a study that has done so. The confounder applies to RGN measured in serum. It does not apply to RGN measured in tumor tissue, and it is there that the clinical associations have been developed. Tissue RGN transcript level has been related to survival across several cancer types, and in lung squamous cell carcinoma it has additionally been related to immune cell infiltration and to a predicted response to immune checkpoint blockade. These analyses are retrospective and derived from public transcriptomic cohorts, so they establish association rather than clinical utility; none has been tested prospectively, and protein-level assays suitable for routine use have not been developed. Tissue RGN is nevertheless the more tractable of the two measurements at present, because it is not subject to the hepatic contribution that confounds the circulating protein.

### 7.6. Effects on the Non-Malignant Compartment of the TME Are Untested

The TME comprises cancer-associated fibroblasts, immune cells, endothelial cells, and matrix, and extracellular RGN is proposed to act on this ecosystem as a whole. The supporting data, however, are derived almost entirely from cancer cell lines in monoculture.

This gap is not explained by an inability of RGN to act on non-malignant cells. Exogenous RGN suppresses the differentiation and mineralization of osteoblastic MCT3T3-E1 cells and reduces Runx2 and alkaline phosphatase transcripts, while leaving cell number unchanged [64]; it stimulates RANKL-induced osteoclastogenesis in RAW264.7 cells [65]; and it suppresses osteoblastogenesis while stimulating adipogenesis in mouse bone marrow culture [66]. Non-transformed cells are therefore responsive to extracellular RGN.

The responses observed in these cells are not, however, a smaller version of those observed in cancer cells. Two differences bear on any therapeutic proposal. First, exogenous RGN did not reduce osteoblast number [64], whereas suppression of proliferation is the principal effect reported in cancer cells; the antiproliferative activity may therefore be selective, but this has not been examined systematically across lineages. Second, exogenous RGN increases basal and RANKL-induced NF-κB activation in preosteoclastic cells and enhances TNF-α-induced NF-κB activation in preosteoblastic cells [67]—the opposite direction to the reduction in NF-κB activity attributed to RGN in cancer cells. A protein whose effect on a given pathway is reversed between transformed and non-transformed cells cannot be assumed to act uniformly across the TME.

Selectivity also bears on safety. Rats overexpressing regucalcin develop bone loss and hyperlipidemia with age, with elevated serum triglyceride, cholesterol, and free fatty acids and reduced hepatic lipid and glycogen content [67,68]. These animals model sustained intracellular overexpression rather than administration of the protein, so the phenotype does not transfer directly to a therapeutic setting. It does indicate, however, that raising RGN systemically is not without consequence, and that a strategy based on extracellular RGN would require the skeletal and metabolic compartments to be monitored. The tolerability of administered RGN in vivo has not been examined.

The consequence is that the TME-level claim currently rests on an extrapolation, and the one non-malignant lineage that has been examined does not support a simple extension of the cancer cell data. A protein that restrains tumor cells but simultaneously dampens antitumor immunity would produce a net effect that monoculture experiments cannot predict; conversely, a protein that also normalizes activated stroma would exert an activity greater than that inferred from cancer cells alone. Neither possibility can presently be excluded. Co-culture systems, organotypic models, and immunocompetent in vivo models are required before the effect of extracellular RGN on the TME as an integrated system can be stated.

## 8. Therapeutic and Biomarker Implications

The preclinical case for RGN as a therapeutic target is consistent, and the mechanisms underlying it are described in Section 3. This section addresses what follows from that case: how RGN might be delivered, how it behaves in combination with existing agents, and what its measurement in patients would require.

In preclinical experiments, forced expression of the RGN gene or protein introduction therapy has been shown to inhibit the proliferation of various cancer cell lines, including liver [31], breast [32], pancreatic [33], and prostate cancers [53], and arrest the cell cycle at the G1 and G2/M phases. RGN has also demonstrated inhibitory effects on cell proliferation and metastasis and has overcome drug resistance in combination therapy models with existing antitumor drugs (such as gemcitabine) [53]. Any use of circulating RGN as a biomarker, however, must contend with its hepatic origin, which is discussed in Section 7.4.

In cancer treatment research, the integration of advanced delivery systems, such as liposomal nanoparticles, viral vectors, and peptide drugs, is under consideration [6,60]. Liposomes and peptide-modified nanoparticles are anticipated to facilitate selective delivery and enhance their uptake into tumor tissues. However, challenges persist regarding hepatic accumulation, immune clearance, in vivo stability, and target specificity. Consequently, innovations are imperative to achieve efficient tumor targeting and mitigate the side effects. Various modification techniques, such as PEGylation and ligand design, have been developed to address challenges related to in vivo stability, pharmacokinetics, immune responses, and off-target organ distribution. However, clinical trial data on RGN remain limited, and further optimization and research are required for its application.

The preclinical data therefore support continued investigation of RGN as a therapeutic target. Which route—gene delivery or administration of the protein—is the more tractable depends on questions of mechanism that remain open (Section 7).

## 9. Conclusions and Perspectives

RGN is a tumor suppressor whose intracellular activity is supported by consistent data across multiple cancer types (Section 3 and Section 4). The extracellular activity of RGN is a different matter. Recombinant RGN applied to cancer cells reproduces much of the effect seen on intracellular overexpression, and RGN is present outside the cell. What connects these two observations is not known. RGN has no assigned export route; its reported association with EVs has not been tested against current standards; no receptor has been identified; and its effect on the non-malignant compartment of the TME has not been examined. Whether extracellular RGN constitutes a signaling axis within the TME, or is a pharmacologically useful property of a recombinant protein, cannot presently be decided.

The therapeutic potential of RGN is significant and supported by preclinical evidence. Therapeutic strategies, including enforced RGN gene expression and protein introduction, have been shown to inhibit cancer cell proliferation and metastasis in vitro. Furthermore, RGN-based therapies have demonstrated the ability to overcome resistance to existing antitumor drugs, such as gemcitabine, and diminish cancer stem cell properties. This case is not weakened by the gaps set out in Section 7. A protein need not be a physiological signal to be a useful therapeutic agent. But if extracellular RGN proves to be pharmacological rather than physiological, it ceases to be a lead into the biology of the TME, and the two should not be conflated.

The experiments that would decide this are not technically demanding. Density-gradient separation with parallel quantification of RGN in vesicular and non-vesicular fractions, together with protease protection, would establish whether RGN is carried by EVs. Cell-surface binding assays and chemical crosslinking with proteomic identification would identify the proximal target, if one exists. Co-culture systems and immunocompetent in vivo models would show whether the non-malignant cells of the TME respond to extracellular RGN at all. And any proposal to measure RGN in serum requires, at minimum, adjustment for hepatic function. Until these are done, the description of RGN as a signaling molecule of the TME remains an inference drawn from cancer cell monoculture.

## Figures and Tables

**Table 1:** Reported effects of regucalcin expression on cancer cell behavior across cancer types.

Cancer Type	Cell Type	RGN Expression	Function	Ref.
Liver	HepG2	overexpression	up: p21, down: NF-kB, p65, β-catenin	[31]
	HepG2	overexpression	suppression of the growth of cells through suppression of calcium signaling	[14]
	HepG2	exogenous	suppression of colony formation	[41]
	H4-II-E cells	overexpression	promote p53 or Rb mRNA expression	[42]
	H4-II-E cells	overexpression	suppression of cell proliferation	[43]
Breast	MDA-MB-231	exogenous	suppression of proliferation	[14]
	MDA-MB-231	overexpression	suppression of osteoclastogenesis	[32]
Prostate	PNT1A, LNCaP	mediated by extracellular calcium	suppression of cell proliferation, down regulation of Ca^2+^-sensing receptor	[44]
	Transgenic mouse		down regulation of glycolytic metabolism diminished expression of glucose transporter 3 and glycolytic enzyme phosphofructokinase	[45]
	Transgenic mouse	overexpression	promote dormancy, promoted multiple known hallmarks of tumor dormancy including activation of p38 MAPK, decrease in Erk signaling and inhibition of FOXM1 expression	[35]
	PC-3, DU-145	extracellular	inhibition of migration, invasion, and adhesion	[16]
Lung	A549	overexpression	suppressed the proliferation, cell death, and migration	[46]
Colon	RKO cell line	overexpression	suppression of cell proliferation through suppression of AHR signaling	[47]
Brain	Glioblastoma cells		inhibition of metastatic activity, including adhesion, invasion, and migration	[40]
Other	Saos-2 human osteosarcoma cells	overexpression	suppression of colony formation and proliferation	[48]

**Table 2:** Evidentiary status of the principal claims concerning regucalcin in cancer.

Claim	Evidence to Date	Level	Not Yet Established
Intracellular RGN suppresses cancer cell proliferation	Overexpression/knockdown in multiple lines; xenograft	In vitro + in vivo (murine)	Effect of physiological RGN restoration in autochthonous tumors
RGN expression is reduced in human tumors	TCGA/GEO transcriptomic analyses	Human (transcript)	Protein-level confirmation in matched tissue; causality
Extracellular RGN suppresses cancer cell growth and migration	Exogenous recombinant RGN applied to cultured cells	In vitro only	Efficacy in vivo; effective concentration in tissue
RGN is secreted by cells	Detection in serum and conditioned medium	Descriptive	Secretory route; regulated vs. passive release
RGN is an EV cargo	Detection in EV preparations from biofluids	Preliminary (co-isolation)	Orthogonal validation per MISEV2023; vesicular vs. soluble partition
Extracellular RGN engages a cell-surface receptor	Downstream pathway modulation	Inferred	Receptor identity; direct binding
Extracellular RGN acts on the TME as a system	Effects on cancer cells; responsiveness of non-malignant cells in other contexts	Extrapolation	Any direct test on CAFs, immune, or endothelial cells
Serum RGN is a tumor biomarker	Association studies	Proposed	Discrimination from hepatic origin; prospective validation

Levels denote the strongest type of evidence currently available for each claim, not the strength of the effect. In vitro only: cultured-cell data alone; in vitro + in vivo (murine): supported additionally by mouse models; human (transcript): based on human transcriptomic data without protein-level or causal confirmation; descriptive: reported without mechanistic testing; preliminary (co-isolation): based on detection in an EV-enriched fraction without orthogonal validation; inferred: deduced from downstream readouts rather than direct observation; extrapolation: generalized from a different cell type or biological context; proposed: suggested but not directly tested.

## Data Availability

No new data were created or analyzed in this study. Data sharing is not applicable to this article.

## References

[1] Wang L., Wang W., Hu D., Liang Y., Liu Z., Zhong T., Wang X. (2024). Tumor-Derived Extracellular Vesicles Regulate Macrophage Polarization: Role and Therapeutic Perspectives. Front. Immunol..

[2] Lopez K., Lai S.W.T., Lopez Gonzalez E.D.J., Dávila R.G., Shuck S.C. (2023). Extracellular Vesicles: A Dive into Their Role in the Tumor Microenvironment and Cancer Progression. Front. Cell Dev. Biol..

[3] Valadi H., Ekström K., Bossios A., Sjöstrand M., Lee J.J., Lötvall J.O. (2007). Exosome-Mediated Transfer of mRNAs and microRNAs Is a Novel Mechanism of Genetic Exchange between Cells. Nat. Cell Biol..

[4] Raposo G., Stoorvogel W. (2013). Extracellular Vesicles: Exosomes, Microvesicles, and Friends. J. Cell Biol..

[5] Kowal J., Tkach M., Théry C. (2014). Biogenesis and Secretion of Exosomes. Curr. Opin. Cell Biol..

[6] Yamaguchi M. (2023). Regucalcin Is a Potential Regulator in Human Cancer: Aiming to Expand into Cancer Therapy. Cancers.

[7] Harikrishna P., Thomas J., Shende A.M., Bhure S.K. (2017). Calcium Binding Ability of Recombinant Buffalo Regucalcin: A Study Using Circular Dichroism Spectroscopy. Protein J..

[8] Fujita T., Uchida K., Maruyama N. (1992). Purification of Senescence Marker Protein-30 (SMP30) and Its Androgen-Independent Decrease with Age in the Rat Liver. Biochim. Biophys. Acta.

[9] Fujita T., Mandel J.-L., Shirasawa T., Hino O., Shirai T., Maruyama N. (1995). Isolation of cDNA Clone Encoding Human Homologue of Senescence Marker Protein-30 (SMP30) and Its Location on the X Chromosome. Biochim. Biophys. Acta (BBA)-Gene Struct. Expr..

[10] Yamaguchi M. (2013). Suppressive Role of Regucalcin in Liver Cell Proliferation: Involvement in Carcinogenesis. Cell Prolif..

[11] Marques R., Maia C.J., Vaz C., Correia S., Socorro S. (2014). The Diverse Roles of Calcium-Binding Protein Regucalcin in Cell Biology: From Tissue Expression and Signalling to Disease. Cell. Mol. Life Sci..

[12] Li X., Huang Y., Wang P., Song W., Yao Q., Hu Q., Zhou S. (2019). A Mechanism of Regucalcin Knock-down in the Promotion of Proliferation and Movement of Human Cervical Cancer HeLa Cells. Transl. Cancer Res..

[13] Yamaguchi M. (2025). Extracellular Regucalcin: A Potent Suppressor in the Cancer Cell Microenvironment. Cancers.

[14] Yamaguchi M., Murata T., Ramos J.W. (2020). The Calcium Channel Agonist Bay K 8644 Promotes the Growth of Human Liver Cancer HepG2 Cells in Vitro: Suppression with Overexpressed Regucalcin. Mol. Cell Biochem..

[15] Yamaguchi M., Murata T. (2015). Exogenous Regucalcin Suppresses the Proliferation of Human Breast Cancer MDA-MB-231 Bone Metastatic Cells in Vitro. Mol. Med. Rep..

[16] Yamaguchi M., Murata T., Ramos J.W. (2022). Extracellular Regucalcin Suppresses the Growth, Migration, Invasion, and Adhesion of Metastatic Human Prostate Cancer Cells. Oncology.

[17] Arakawa K., Inoue H., Ishigami A., Sato A., Takino Y., Tanaka M., Morimoto H., Takahashi N., Uehara M. (2023). Release of SMP30 in Extracellular Vesicles under Conditions of Ascorbic Acid Deficiency Is Involved with Acute Phase Response in ODS Rat. J. Nutr. Sci. Vitaminol..

[18] Zubiri I., Posada-Ayala M., Benito-Martin A., Maroto A.S., Martin-Lorenzo M., Cannata-Ortiz P., de la Cuesta F., Gonzalez-Calero L., Barderas M.G., Fernandez-Fernandez B. (2015). Kidney Tissue Proteomics Reveals Regucalcin Downregulation in Response to Diabetic Nephropathy with Reflection in Urinary Exosomes. Transl. Res..

[19] Welsh J.A., Goberdhan D.C.I., O’Driscoll L., Buzas E.I., Blenkiron C., Bussolati B., Cai H., Di Vizio D., Driedonks T.A.P., Erdbrügger U. (2024). Minimal Information for Studies of Extracellular Vesicles (MISEV2023): From Basic to Advanced Approaches. J. Extracell. Vesicle.

[20] Laurentino S.S., Correia S., Cavaco J.E., Oliveira P.F., Sousa M.D., Barros A., Socorro S. (2012). Regucalcin, a Calcium-Binding Protein with a Role in Male Reproduction?. Mol. Hum. Reprod..

[21] Danish M., Ahmad R. (2023). Functional Pleiotropy of Calcium Binding Protein Regucalcin in Signaling and Diseases. Cell. Signal..

[22] Yamaguchi M. (2013). The Anti-Apoptotic Effect of Regucalcin Is Mediated through Multisignaling Pathways. Apoptosis.

[23] Laurentino S.S., Correia S., Cavaco J.E., Oliveira P.F., Rato L., Sousa M., Barros A., Socorro S. (2011). Regucalcin Is Broadly Expressed in Male Reproductive Tissues and Is a New Androgen-Target Gene in Mammalian Testis. Reproduction.

[24] Misawa H., Yamaguchi M. (2000). The Gene of Ca2+-Binding Protein Regucalcin Is Highly Conserved in Vertebrate Species. Int. J. Mol. Med..

[25] Yamaguchi M. (2009). Novel Protein RGPR-P117: Its Role as the Regucalcin Gene Transcription Factor. Mol. Cell Biochem..

[26] Singh T., Banerjee P., Uditi, Kumari S., Chopra A., Singh N., Qamar I. (2022). Expression of Regucalcin, a Calcium-Binding Protein Is Regulated by Hypoxia-Inducible Factor-1α. Life Sci..

[27] Yamaguchi M. (2014). Regulatory Role of Regucalcin in Heart Calcium Signaling: Insight into Cardiac Failure (Review). Biomed. Rep..

[28] Vaz C.V., Marques R., Maia C.J., Socorro S. (2015). Aging-Associated Changes in Oxidative Stress, Cell Proliferation, and Apoptosis Are Prevented in the Prostate of Transgenic Rats Overexpressing Regucalcin. Transl. Res..

[29] Fujita T., Shirasawa T., Uchida K., Maruyama N. (1996). Gene Regulation of Senescence Marker Protein-30 (SMP30): Coordinated up-Regulation with Tissue Maturation and Gradual down-Regulation with Aging. Mech. Ageing Dev..

[30] Jung K.J., Lee E.K., Kim S.J., Song C.W., Maruyama N., Ishigami A., Kim N.D., Im D.-S., Yu B.P., Chung H.Y. (2015). Anti-Inflammatory Activity of SMP30 Modulates NF-κB through Protein Tyrosine Kinase/Phosphatase Balance. J. Mol. Med..

[31] Yamaguchi M., Osuka S., Weitzmann M.N., El-Rayes B.F., Shoji M., Murata T. (2016). Prolonged Survival in Hepatocarcinoma Patients with Increased Regucalcin Gene Expression: HepG2 Cell Proliferation Is Suppressed by Overexpression of Regucalcin in Vitro. Int. J. Oncol..

[32] Yamaguchi M., Osuka S., Weitzmann M.N., Shoji M., Murata T. (2016). Increased Regucalcin Gene Expression Extends Survival in Breast Cancer Patients: Overexpression of Regucalcin Suppresses the Proliferation and Metastatic Bone Activity in MDA-MB-231 Human Breast Cancer Cells in Vitro. Int. J. Oncol..

[33] Yamaguchi M., Osuka S., Murata T., Ramos J.W. (2021). Progression-Free Survival of Prostate Cancer Patients Is Prolonged with a Higher Regucalcin Expression in the Tumor Tissues: Overexpressed Regucalcin Suppresses the Growth and Bone Activity in Human Prostate Cancer Cells. Transl. Oncol..

[34] Li X., Huang Y., Guo S., Xie M., Bin X., Shi M., Chen A., Chen S., Wu F., Hu Q. (2019). Exogenous Regucalcin Negatively Regulates the Progression of Cervical Adenocarcinoma. Oncol. Lett..

[35] Sharma S., Pei X., Xing F., Wu S.-Y., Wu K., Tyagi A., Zhao D., Deshpande R., Ruiz M.G., Singh R. (2021). Regucalcin Promotes Dormancy of Prostate Cancer. Oncogene.

[36] Yamaguchi M., Osuka S., Hankinson O., Murata T. (2018). Prolonged Survival of Renal Cancer Patients Is Concomitant with a Higher Regucalcin Gene Expression in Tumor Tissues: Overexpression of Regucalcin Suppresses the Growth of Human Renal Cell Carcinoma Cells in Vitro. Int. J. Oncol..

[37] Shao C., Guo K., Xu L., Zhang Y., Duan H., Feng Y., Pan M., Lu D., Ren X., Ganti A.K. (2021). Senescence Marker Protein 30 Inhibits Tumor Growth by Reducing HDAC4 Expression in Non-Small Cell Lung Cancer. Transl. Lung Cancer Res..

[38] Wu B., Crampton S.P., Hughes C.C.W. (2007). Wnt Signaling Induces Matrix Metalloproteinase Expression and Regulates T Cell Transmigration. Immunity.

[39] Yamaguchi M. (2015). The Potential Role of Regucalcin in Kidney Cell Regulation: Involvement in Renal Failure (Review). Int. J. Mol. Med..

[40] Yamaguchi M., Shimokawa N., Murata T. (2025). Extracellular Regucalcin Reveals Anti-Cancer Activity by Suppressing Cell Growth and Metastatic Activity by Blocking EGF Signaling Pathway in Human Glioblastoma Cells in Vitro. Cell. Signal..

[41] Yamaguchi M., Murata T. (2018). Exogenous Regucalcin Suppresses the Growth of Human Liver Cancer HepG2 Cells in Vitro. Oncol. Rep..

[42] Tsurusaki Y., Yamaguchi M. (2004). Role of Regucalcin in Liver Nuclear Function: Binding of Regucalcin to Nuclear Protein or DNA and Modulation of Tumor-Related Gene Expression. Int. J. Mol. Med..

[43] Yamaguchi M., Daimon Y. (2005). Overexpression of Regucalcin Suppresses Cell Proliferation in Cloned Rat Hepatoma H4-II-E Cells: Involvement of Intracellular Signaling Factors and Cell Cycle-related Genes. J. Cell. Biochem..

[44] Vaz C.V., Rodrigues D.B., Socorro S., Maia C.J. (2015). Effect of Extracellular Calcium on Regucalcin Expression and Cell Viability in Neoplastic and Non-Neoplastic Human Prostate Cells. Biochim. Biophys. Acta (BBA)-Mol. Cell Res..

[45] Vaz C.V., Marques R., Cardoso H.J., Maia C.J., Socorro S. (2016). Suppressed Glycolytic Metabolism in the Prostate of Transgenic Rats Overexpressing Calcium-Binding Protein Regucalcin Underpins Reduced Cell Proliferation. Transgen. Res..

[46] Yamaguchi M., Osuka S., Shoji M., Weitzmann M.N., Murata T. (2017). Survival of Lung Cancer Patients Is Prolonged with Higher Regucalcin Gene Expression: Suppressed Proliferation of Lung Adenocarcinoma A549 Cells in Vitro. Mol. Cell Biochem..

[47] Yamaguchi M., Hankinson O. (2019). 2,3,7,8-tetrachlorodibenzo-p-dioxin Suppresses the Growth of Human Colorectal Cancer Cells in Vitro: Implication of the Aryl Hydrocarbon Receptor Signaling. Int. J. Oncol..

[48] Yamaguchi M., Murata T. (2020). Overexpression of Regucalcin Suppresses the Growth of Human Osteosarcoma Cells in Vitro: Repressive Effect of Extracellular Regucalcin. Cancer Investig..

[49] Fonseca L.R.S., Carreira R.J.P., Feijó M., Cavaco J.E.B., Cardoso H.J., Vaz C.V., Figueira M.I., Socorro S. (2024). Downregulated Regucalcin Expression Induces a Cancer-like Phenotype in Non-Neoplastic Prostate Cells and Augments the Aggressiveness of Prostate Cancer Cells: Interplay with the G Protein-Coupled Oestrogen Receptor?. Cancers.

[50] Yamaguchi M. (2011). Regucalcin and Cell Regulation: Role as a Suppressor Protein in Signal Transduction. Mol. Cell Biochem..

[51] Nitschkowski D., Marwitz S., Kotanidou S.A., Reck M., Kugler C., Rabe K.F., Ammerpohl O., Goldmann T. (2019). Live and Let Die: Epigenetic Modifications of Survivin and Regucalcin in Non-Small Cell Lung Cancer Tissues Contribute to Malignancy. Clin. Epigenet..

[52] Liao Y., Cheng W., Mou R., Li X., Jia Y. (2023). RGN as a Prognostic Biomarker with Immune Infiltration and ceRNA in Lung Squamous Cell Carcinoma. Sci. Rep..

[53] Yamaguchi M., Murata T. (2015). Suppressive Effects of Exogenous Regucalcin on the Proliferation of Human Pancreatic Cancer MIA PaCa-2 Cells in Vitro. Int. J. Mol. Med..

[54] Margolis L., Sadovsky Y. (2019). The Biology of Extracellular Vesicles: The Known Unknowns. PLoS Biol..

[55] Johnsen K.B., Gudbergsson J.M., Andresen T.L., Simonsen J.B. (2019). What Is the Blood Concentration of Extracellular Vesicles? Implications for the Use of Extracellular Vesicles as Blood-Borne Biomarkers of Cancer. Biochim. Biophys. Acta (BBA)-Rev. Cancer.

[56] Jacob V., De Berny Q., Brazier F., Presne C., Lion J., Ouled-Haddou H., Metzinger-Le Meuth V., Choukroun G., Metzinger L., Guillaume N. (2025). Quantification of Urine and Plasma Levels of Extracellular Vesicles in a Cohort of Kidney Transplant Recipients and Chronic Kidney Disease Patients. IJMS.

[57] Itzel C., Eduardo M., Velia R., Mireya C., Alfredo H., Pilar R.M.D., Gabriela A. (2025). Salivary Extracellular Vesicles Separation: Analysis of Ultracentrifugation—Based Protocols. Oral Dis..

[58] Sandau U.S., Magaña S.M., Costa J., Nolan J.P., Ikezu T., Vella L.J., Jackson H.K., Moreira L.R., Palacio P.L., Hill A.F. (2024). Recommendations for Reproducibility of Cerebrospinal Fluid Extracellular Vesicle Studies. J. Extracell. Vesicle.

[59] Ramirez M.I., Amorim M.G., Gadelha C., Milic I., Welsh J.A., Freitas V.M., Nawaz M., Akbar N., Couch Y., Makin L. (2018). Technical Challenges of Working with Extracellular Vesicles. Nanoscale.

[60] Spicer C.D., Jumeaux C., Gupta B., Stevens M.M. (2018). Peptide and Protein Nanoparticle Conjugates: Versatile Platforms for Biomedical Applications. Chem. Soc. Rev..

[61] Chitti S.V., Gummadi S., Kang T., Shahi S., Marzan A.L., Nedeva C., Sanwlani R., Bramich K., Stewart S., Petrovska M. (2024). Vesiclepedia 2024: An Extracellular Vesicles and Extracellular Particles Repository. Nucleic Acids Res..

[62] Chitti S.V., Kang T., Fonseka P., Marzan A.L., Stewart S., Shahi S., Bramich K., Ang C., Pathan M., Gummadi S. (2023). Proteomic Analysis of the Small Extracellular Vesicles and Soluble Secretory Proteins from Cachexia Inducing and Non-inducing Cancer Cells. Proteomics.

[63] Yamaguchi M., Mori S., Kato S. (1988). Calcium-Binding Protein Regucalcin Is an Activator of (Ca2+-Mg2+)-Adenosine Triphosphatase in the Plasma Membranes of Rat Liver. Chem. Pharm. Bull..

[64] Otomo Y., Yamaguchi M. (2006). Regulatory Effect of Exogenous Regucalcin on Cell Function in Osteoblastic MC3T3-E1 Cells: Involvement of Intracellular Signaling Factor. Int. J. Mol. Med..

[65] Yamaguchi M., Neale Weitzmann M., Murata T. (2012). Exogenous Regucalcin Stimulates Osteoclastogenesis and Suppresses Osteoblastogenesis through NF-κB Activation. Mol. Cell Biochem..

[66] Yamaguchi M., Weitzmann M.N., Baile C.A., Murata T. (2012). Exogenous Regucalcin Suppresses Osteoblastogenesis and Stimulates Adipogenesis in Mouse Bone Marrow Culture. Integr. Biol..

[67] Yamaguchi M., Nakagawa T. (2007). Change in Lipid Components in the Adipose and Liver Tissues of Regucalcin Transgenic Rats with Increasing Age: Suppression of Leptin and Adiponectin Gene Expression. Int. J. Mol. Med..

[68] Yamaguchi M. (2010). Regucalcin and Metabolic Disorders: Osteoporosis and Hyperlipidemia Are Induced in Regucalcin Transgenic Rats. Mol. Cell Biochem..

